# Supplementary material for: The Evolution and Characterization of the RNA Interference Pathways in Lophotrochozoa
Source: Genome Biol Evol. 2024 May 7;16(5):evae098. doi: 10.1093/gbe/evae098 (PMC11114477; doi:10.1093/gbe/evae098)
Supplement: evae098_Supplementary_Data [file evae098_supplementary_data.zip › Supplementary_materials.zip!/selected_miRNAs/NoGe_selected_miRNAs/mirdeep_pdf_files/NMRB01000231.1_Notospermus_geniculatus_strain_Ushimado_scaffold231,_whole_genome_shotgun_sequence_7811.pdf]

Provisional ID	:	NMRB01000231.1_Notospermus_geniculatus_strain_Ushimado_scaffold231,_whole_genome_shotgun_sequence_7811
Score total	:	55280.5
Score for star read(s)	:	3.9
Score for read counts	:	55271.8
Score for mfe	:	1.8
Score for randfold	:	0
Score for cons. seed	:	3
Total read count	:	108425
Mature read count	:	108131
Loop read count	:	0
Star read count	:	294

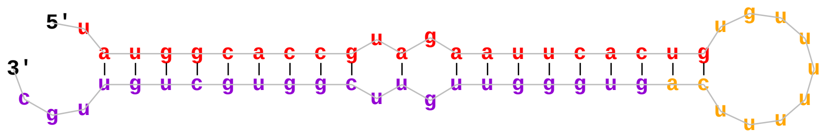

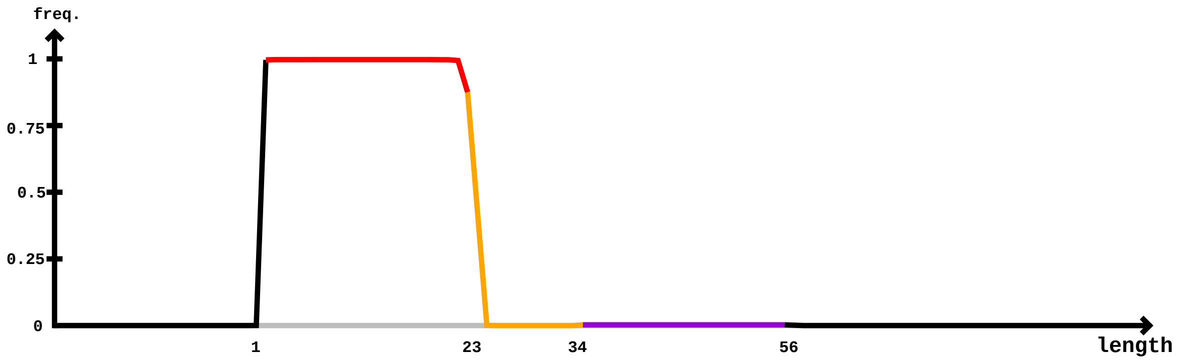

Mature Star

```
5'- ucaucucugcuuacaagagcuauggcaccguagaauucacuguguuuuuuucaguggguuguucggugcuguugcuuuugauguagauagcugcuguugucacuugugauuc  -3'   obs
    ucaucucugcuuacaagagcuauggcaccguagaauucacuguguuuuuuucaguggguuguucggugcuguugcuuuugauguagauagcugcuguugucacuugugauuc         exp
    .......(((.(.(((((((.(((((((((.(.(((((((((.........))))))))).).))))))))).))))))).).)))((((((....))))))..........   reads   mm   sample
    .caucucugcuuacaagagc............................................................................................   1       0    Fm1
    ...................cuauggcaccguagaauucacu.......................................................................   1       0    Fm1
    ....................uauggcaccguagaauuc..........................................................................   3       0    Fm1
    ....................uauggcaccguagaauucac........................................................................   8       0    Fm1
    ....................uauggcaccgAagaauucacu.......................................................................   3       1    Fm1
    ....................uauggcaccguagaauucacG.......................................................................   2       1    Fm1
    ....................uauggcaccguagaauuUacu.......................................................................   5       1    Fm1
    ....................uauggcGccguagaauucacu.......................................................................   4       1    Fm1
    ....................uauggcaccguagaaCucacu.......................................................................   2       1    Fm1
    ....................uauggcaccgGagaauucacu.......................................................................   4       1    Fm1
    ....................uauggcaccguagaauCcacu.......................................................................   1       1    Fm1
    ....................uauggcaccgCagaauucacu.......................................................................   2       1    Fm1
    ....................uauggcacUguagaauucacu.......................................................................   1       1    Fm1
    ....................uauggcaccguagaauuAacu.......................................................................   10      1    Fm1
    ....................uUuggcaccguagaauucacu.......................................................................   2       1    Fm1
    ....................uauggcaccguagaauGcacu.......................................................................   3       1    Fm1
    ....................uauggcaccguagaauucacu.......................................................................   1315    0    Fm1
    ....................uaugUcaccguagaauucacu.......................................................................   2       1    Fm1
    ....................uauggcaccguaCaauucacu.......................................................................   1       1    Fm1
    ....................uauggcaccguagaauAcacu.......................................................................   2       1    Fm1
    ....................uauggcaccUuagaauucacu.......................................................................   1       1    Fm1
    ....................uauggcaUcguagaauucacu.......................................................................   1       1    Fm1
    ....................uauggcaccguagaauuGacu.......................................................................   3       1    Fm1
    ....................uauggcaccguagaauucUcug......................................................................   1       1    Fm1
    ....................uaugUcaccguagaauucacug......................................................................   1       1    Fm1
    ....................uauggcaccguagaauucacuC......................................................................   8       1    Fm1
    ....................uUuggcaccguagaauucacug......................................................................   1       1    Fm1
    ....................uauggcacUguagaauucacug......................................................................   1       1    Fm1
    ....................uaCggcaccguagaauucacug......................................................................   2       1    Fm1
    ....................uauggcaccguagaauGcacug......................................................................   8       1    Fm1
    ....................uauggcaccguagaauucaGug......................................................................   1       1    Fm1
    ....................uauggcaccguagaauCcacug......................................................................   7       1    Fm1
    ....................uauggcaccguagaauucGcug......................................................................   1       1    Fm1
```

Mature **Star**

ucaucucugcuuacaagagcuauggcaccguagaauucacuguguuuuuuucaguggguuguucggugcuguugcuuuugauguagauagcugcuguugucacuugugauuc			
....................uauggcaccgAagaauucacug..	3	1	Fm1
....................uauggcaccguagaauAcacug..	8	1	Fm1
....................uauggcaccguagUauucacug..	1	1	Fm1
....................uauggcaccguagaauucacGg..	12	1	Fm1
....................uauggcaccguagaauuUacug..	13	1	Fm1
....................uauggcaccguCgaauucacug..	3	1	Fm1
....................uGuggcaccguagaauucacug..	1	1	Fm1
....................uauggcaccguagaauuAacug..	22	1	Fm1
....................uauggcCccguagaauucacug..	1	1	Fm1
....................uauggcaccguagCauucacug..	1	1	Fm1
....................uauggGaccguagaauucacug..	1	1	Fm1
....................uauUgcaccguagaauucacug..	2	1	Fm1
....................uauggcaccguagaauuGacug..	5	1	Fm1
....................uauggcaccAuagaauucacug..	1	1	Fm1
....................uauggcaccgGagaauucacug..	4	1	Fm1
....................uauggUaccguagaauucacug..	1	1	Fm1
....................uauggcaccguGgaauucacug..	2	1	Fm1
....................uauggcaccguagaauucacuA..	9	1	Fm1
....................uauggcaccgCagaauucacug..	2	1	Fm1
....................uauggcaccguagaauucacug..	2733	0	Fm1
....................uauggcaccguagaauucacuU..	4	1	Fm1
....................uauggcaccguagaGuucacug..	1	1	Fm1
....................uauggcaccguagaauuAacugu...	1	1	Fm1
....................uaCggcaccguagaauucacugu...	1	1	Fm1
....................uauggcaccUuagaauucacugu...	1	1	Fm1
....................uauggcaccguagaauucacugA...	56	1	Fm1
....................uauggcaccguagaauucacugG...	3	1	Fm1
....................uauggcaccguagaauucUcugu...	1	1	Fm1
....................uauggcaccguagaauuUacugu...	1	1	Fm1
....................uauggcaccguagaauuGacugu...	2	1	Fm1
....................uauggcaccguGgaauucacugu...	1	1	Fm1
....................uauggUaccguagaauucacugu...	1	1	Fm1
....................uauggcaccguagaauucacugC...	3	1	Fm1
....................uauggcaccguagaauucacugu...	173	0	Fm1
....................uauggcaccguagaauucacuguA..	1	1	Fm1
....................uauggcaccguagaauucacuguU..	3	1	Fm1
.....................auggcaccguagaauucacug..	1	0	Fm1
......................uggcaccguagaauucacu...	1	0	Fm1
....guggguuguucggugcCguugc.....................................	1	1	Fm1
....guggguuguucggugcuguugc.....................................	10	0	Fm1
...................cuauggcaccguagaauucacu...	6	0	Fm2
....................uauggcaccguagaauuA..	9	1	Fm2
....................uaGggcaccguagaauucacu...	1	1	Fm2
....................uauggcacUguagaauucacu...	36	1	Fm2
....................uauggcaccguagaauucacu...	2311	0	Fm2
....................uauggcaccguagaauuUacu...	9	1	Fm2
....................uauggcaccguagaauCcacu...	5	1	Fm2
....................uauggcaccguagGauucacu...	1	1	Fm2
....................Nauggcaccguagaauucacu...	1	1	Fm2
....................uauggcaccguagaauucacA...	1	1	Fm2
....................uauggcaccguagUauucacu...	1	1	Fm2
....................uauggcaccguagaauuGacu...	11	1	Fm2
....................uauggUaccguagaauucacu...	1	1	Fm2
....................uauggcaccgGagaauucacu...	1	1	Fm2
....................uauggcaccgAagaauucacu...	4	1	Fm2
....................uauggcaccguagaaCucacu...	4	1	Fm2
....................uaCggcaccguagaauucacu...	1	1	Fm2
....................uauggcaccguagaauuAacu...	23	1	Fm2
....................uauggcaccguGgaauucacu...	2	1	Fm2
....................uauAgcaccguagaauucacu...	2	1	Fm2
....................uauggcaccguagaauucacG...	9	1	Fm2
....................uCuggcaccguagaauucacu...	2	1	Fm2
....................uauggcaccAuagaauucacu...	1	1	Fm2
....................Cauggcaccguagaauucacu...	4	1	Fm2
....................Aauggcaccguagaauucacu...	1	1	Fm2
....................uauggcaccguagaauAcacu...	5	1	Fm2
....................uauggNaccguagaauucacu...	1	1	Fm2
....................uauggcaccguagaauGcacu...	2	1	Fm2
....................uauggcaccgCagaauucacu...	4	1	Fm2

```
                    Mature                Star
ucaucucugcuuacaagagcuauggcaccguagaauucacuguguuuuuuucagugguuguucggugcuguugcuuuugauguagauagcugcuguugucacuugugauuc
....................uGuggcaccguagaauucacu......................................................................    2     1     Fm2
....................uUuggcaccguagaauucacug.....................................................................    1     1     Fm2
....................uauggcacUguagaauucacug.....................................................................    4     1     Fm2
....................uauggcaccguaUaauucacug.....................................................................    1     1     Fm2
....................uauggcacAguagaauucacug.....................................................................    1     1     Fm2
....................uauggcaccguCgaauucacug.....................................................................    3     1     Fm2
....................uauggcaccguagaGuucacug.....................................................................    1     1     Fm2
....................uauggcaccguagaauuUacug.....................................................................    23    1     Fm2
....................uauUgcaccguagaauucacug.....................................................................    1     1     Fm2
....................uauggcaccguagaauuGacug.....................................................................    12    1     Fm2
....................uauggcaccguagaauucUcug.....................................................................    1     1     Fm2
....................uauggcaUcguagaauucacug.....................................................................    1     1     Fm2
....................uauggcaccguagaauAcacug.....................................................................    5     1     Fm2
....................uaugAcaccguagaauucacug.....................................................................    1     1     Fm2
....................uauggcaccguagaauucacCg.....................................................................    1     1     Fm2
....................uauggcaccguagaauucacGg.....................................................................    4     1     Fm2
....................uauggcaccguagaauucCcug.....................................................................    1     1     Fm2
....................uauggcaccguagaauuAacug.....................................................................    19    1     Fm2
....................Nauggcaccguagaauucacug.....................................................................    3     1     Fm2
....................uauggcaccguagaauucacuU.....................................................................    1     1     Fm2
....................uCuggcaccguagaauucacug.....................................................................    2     1     Fm2
....................uauggcaccgAagaauucacug.....................................................................    7     1     Fm2
....................uauggcUccguagaauucacug.....................................................................    1     1     Fm2
....................uauggcaccguagaauucacuA.....................................................................    28    1     Fm2
....................uauggcaccguagaauGcacug.....................................................................    8     1     Fm2
....................uauggcaccguagaauCcacug.....................................................................    15    1     Fm2
....................uauggcaccguagaauucacug.....................................................................    2910  0     Fm2
....................uauggcNccguagaauucacug.....................................................................    1     1     Fm2
....................uauggcGccguagaauucacug.....................................................................    1     1     Fm2
....................uGuggcaccguagaauucacug.....................................................................    2     1     Fm2
....................uauggcaccgGagaauucacug.....................................................................    2     1     Fm2
....................uauggcaccguUgaauucacug.....................................................................    1     1     Fm2
....................uauggcaccgCagaauucacug.....................................................................    5     1     Fm2
....................uauggcaccguagaauucacugC....................................................................    14    1     Fm2
....................uauggcaccguagaauucacugu....................................................................    293   0     Fm2
....................uauggcaccguagaauuUacugu....................................................................    1     1     Fm2
....................uauggcaccgCagaauucacugu....................................................................    1     1     Fm2
....................uauggcaccguagaauucacugG....................................................................    41    1     Fm2
....................uauggcaccguagGauucacugu....................................................................    1     1     Fm2
....................uauggcaccguagaauuGacugu....................................................................    1     1     Fm2
....................uauggcaccguagaauGcacugu....................................................................    1     1     Fm2
....................uauggcaccguagaauucacugA....................................................................    171   1     Fm2
....................uauggcaccAuagaauucacugu....................................................................    1     1     Fm2
....................uauggcaccguagaauucacuguU...................................................................    12    1     Fm2
....................uauggcaccguagaauucacugug...................................................................    1     0     Fm2
....................uauggcaccguagaauucacuguA...................................................................    8     1     Fm2
.....................auggcaccguagaauucacu......................................................................    2     0     Fm2

..................cuauggcaccguagaauucacu.......................................................................    1     0     Fm3
..................cuauggcaccguagaauucacug......................................................................    1     0     Fm3
....................uauggcaccguagaauuA.........................................................................    4     1     Fm3
....................uauggcaccguagaauuc.........................................................................    4     0     Fm3
....................uauggcaccguagaauuca........................................................................    4     0     Fm3
....................uauggcaccguagaauucaA.......................................................................    1     1     Fm3
....................uauggcaccguagaauucac.......................................................................    15    0     Fm3
....................uauggcaccguagaauuUac.......................................................................    1     1     Fm3
....................uauggcaccguagaauucacu......................................................................    547   0     Fm3
....................uaugAcaccguagaauucacu......................................................................    1     1     Fm3
....................uauggcGccguagaauucacu......................................................................    2     1     Fm3
....................uauggcaccgCagaauucacu......................................................................    1     1     Fm3
....................uauggcaccguagaauAcacu......................................................................    4     1     Fm3
....................uauggcaccguagaauuGacu......................................................................    4     1     Fm3
....................uauggcacUguagaauucacu......................................................................    6     1     Fm3
....................uauggcaccguagaauuAacu......................................................................    6     1     Fm3
....................uauggcUccguagaauucacu......................................................................    2     1     Fm3
....................uauggcaccguagaauuUacu......................................................................    4     1     Fm3
....................uauggcaccguagaauGcacu......................................................................    1     1     Fm3
....................uauggcaccguagCauucacug.....................................................................    1     1     Fm3
....................uaugUcaccguagaauucacug.....................................................................    1     1     Fm3
....................uauggUaccguagaauucacug.....................................................................    4     1     Fm3
```

```
                    Mature                         Star
ucaucucugcuuacaagagcuauggcaccguagaauucacuguguuuuuuucaguggguuguucggugcuguugcuuuugauguagauagcugcuguugucacuugugauuc
....................uauggcaccguagaauuAacug......................................................................  10    1  Fm3
....................uauggcaccguCgaauucacug......................................................................  2     1  Fm3
....................uauggcacUguagaauucacug......................................................................  3     1  Fm3
....................uauggcaccguagaauGcacug......................................................................  5     1  Fm3
....................uauggcaccguagaauucGcug......................................................................  1     1  Fm3
....................uauggcGccguagaauucacug......................................................................  3     1  Fm3
....................uauggcaccgGagaauucacug......................................................................  1     1  Fm3
....................uauggcaccguagaauucacAg......................................................................  1     1  Fm3
....................Cauggcaccguagaauucacug......................................................................  1     1  Fm3
....................uauggcaccguagaauucacuU......................................................................  1     1  Fm3
....................uauggcaccguagaauuGacug......................................................................  5     1  Fm3
....................uauggcaccguagaaCucacug......................................................................  1     1  Fm3
....................Nauggcaccguagaauucacug......................................................................  1     1  Fm3
....................uauggcaccguagaauucacug......................................................................  1960  0  Fm3
....................uauggcaccguagaauuUacug......................................................................  11    1  Fm3
....................uauggcaccAuagaauucacug......................................................................  1     1  Fm3
....................uauggcaccguagGauucacug......................................................................  1     1  Fm3
....................uauggcaccguagUauucacug......................................................................  1     1  Fm3
....................uauggcaccguagaauAcacug......................................................................  5     1  Fm3
....................uauggcaccgAagaauucacug......................................................................  7     1  Fm3
....................uaGggcaccguagaauucacug......................................................................  1     1  Fm3
....................uauggcaAcguagaauucacug......................................................................  2     1  Fm3
....................uaAggcaccguagaauucacug......................................................................  2     1  Fm3
....................uauggcaccguagaCuucacug......................................................................  1     1  Fm3
....................uauggcaccguagaauCcacug......................................................................  6     1  Fm3
....................uauggcaccguagaauucacuA......................................................................  6     1  Fm3
....................uauggcaccguaUaauucacug......................................................................  1     1  Fm3
....................uauggcaccguagaauucacugu.....................................................................  168   0  Fm3
....................uauggcacUguagaauucacugu.....................................................................  2     1  Fm3
....................uauggcaccguagaauucacugA.....................................................................  26    1  Fm3
....................Nauggcaccguagaauucacugu.....................................................................  1     1  Fm3
....................uauggcaccguagaauucacuguA....................................................................  1     1  Fm3
....................uauggcaccguagaauucacuguU....................................................................  8     1  Fm3
.....................auggcaccguagaauucacu.......................................................................  1     0  Fm3
.....................auggcaccguagaauucacug......................................................................  4     0  Fm3
.....................auggcaccguagaauucacuU......................................................................  1     1  Fm3
.....................auggcaccguagaauucacugA.....................................................................  1     1  Fm3
.....................auggcaccguagaauucacuguA....................................................................  1     1  Fm3
.....................................................guggguuguucggugcuguug......................................  3     0  Fm3
.....................................................guggguuguucggugcuguugc.....................................  3     0  Fm3

...................cuauggcaccguagaauucacug......................................................................  1     0  Mm1
...................cuauggcaccguCgaauucacug......................................................................  14    1  Mm1
....................uauggUaccguagaauucac........................................................................  1     1  Mm1
....................uauggcaccguCgaauucac........................................................................  22    1  Mm1
....................uauggcaccguagaauucac........................................................................  11    0  Mm1
....................uauggcaccgGagaauucacu.......................................................................  3     1  Mm1
....................uauggcaccguagaauuUacu.......................................................................  2     1  Mm1
....................uaGggcaccguagaauucacu.......................................................................  1     1  Mm1
....................uauggcaccguagaauCcacu.......................................................................  4     1  Mm1
....................uauggcaccguagaauuAacu.......................................................................  10    1  Mm1
....................uauggcaccguagaauuGacu.......................................................................  3     1  Mm1
....................uauggcNccguagaauucacu.......................................................................  2     1  Mm1
....................Nauggcaccguagaauucacu.......................................................................  1     1  Mm1
....................uauggcaccguagaauGcacu.......................................................................  4     1  Mm1
....................uauggcaccguagaauAcacu.......................................................................  4     1  Mm1
....................uauggcaccguagaauucacu.......................................................................  837   0  Mm1
....................uaAggcaccguagaauucacu.......................................................................  1     1  Mm1
....................uauggcaccgAagaauucacu.......................................................................  3     1  Mm1
....................uGuggcaccguagaauucacu.......................................................................  1     1  Mm1
....................uauggcaccguCgaauucacu.......................................................................  738   1  Mm1
....................uauggcaccguagaauucUcug......................................................................  1     1  Mm1
....................uauggcaccguGgaauucacug......................................................................  4     1  Mm1
....................uauggcaccgAagaauucacug......................................................................  4     1  Mm1
....................uauggcaccguagaaCucacug......................................................................  1     1  Mm1
....................uauggcaccguagaauuGacug......................................................................  6     1  Mm1
....................uauggcaccguagaauuUacug......................................................................  6     1  Mm1
....................uauggcaUcguagaauucacug......................................................................  1     1  Mm1
....................uauggcaccguagGauucacug......................................................................  6     1  Mm1
....................uauggcacUguagaauucacug......................................................................  8     1  Mm1
```

```
                    Mature                        Star
ucaucucugcuuacaagagcuauggcaccguagaauucacuguguuuuuuucaguggguuguucggugcuguugcuuuugauguagauagcugcuguugucacuugugauuc
....................uCuggcaccguagaauucacug......................................................................   1       1   Mm1
....................uauggcGccguagaauucacug......................................................................   1       1   Mm1
....................uauggcaccguagaCuucacug......................................................................   1       1   Mm1
....................uauggcaccguagaauucacuA......................................................................   1       1   Mm1
....................uauggcaccgGagaauucacug......................................................................   4       1   Mm1
....................uauggcaccAuagaauucacug......................................................................   1       1   Mm1
....................uauggcaccguagaauucacuU......................................................................   2       1   Mm1
....................uauggcaccguCgaauucacug......................................................................   57115   1   Mm1
....................uaGggcaccguagaauucacug......................................................................   2       1   Mm1
....................uauggcaccguagaauCcacug......................................................................   5       1   Mm1
....................Nauggcaccguagaauucacug......................................................................   2       1   Mm1
....................uauggcaccguUgaauucacug......................................................................   12      1   Mm1
....................uauggcaccguagaauuAacug......................................................................   8       1   Mm1
....................uauggcaccguagaauucacug......................................................................   1779    0   Mm1
....................uauggcaccgCagaauucacug......................................................................   2       1   Mm1
....................uauggcaccguagaauucacGg......................................................................   1       1   Mm1
....................uauggcaccguagaauAcacug......................................................................   3       1   Mm1
....................uauggcaccguagaauGcacug......................................................................   3       1   Mm1
....................uGuggcaccguagaauucacugu.....................................................................   1       1   Mm1
....................uauggcaccgCagaauucacugu.....................................................................   1       1   Mm1
....................uauggcaccguCgaauucacugu.....................................................................   1172    1   Mm1
....................uauggcaccguagaauucacugA.....................................................................   86      1   Mm1
....................uauggcaccguagaauucacugu.....................................................................   155     0   Mm1
....................uauggcaccguagaauuGacugu.....................................................................   1       1   Mm1
....................uauggcaccgAagaauucacugu.....................................................................   1       1   Mm1
....................uauggcaccguagaauuAacugu.....................................................................   2       1   Mm1
....................uauggcaccgGagaauucacugu.....................................................................   1       1   Mm1
....................uauggcaccguagaauucacugG.....................................................................   5       1   Mm1
....................uauggcaccguagaauuUacugu.....................................................................   2       1   Mm1
....................uauggcaccguagaauucacuguU....................................................................   6       1   Mm1
....................uauggcaccguagaauucacugAg....................................................................   2       1   Mm1
.....................auggcaccguagaauucacu.......................................................................   1       0   Mm1
.....................auggcaccguCgaauucacug......................................................................   25      1   Mm1
......................uggcaccguCgaauucacug......................................................................   7       1   Mm1
.........................caccguCgaauucacugu.....................................................................   7       1   Mm1
....................................................guggguNguucggugcuguugc......................................   1       1   Mm1
....................................................guggguuguucggugcuguugc......................................   166     0   Mm1
....................................................guggguuguucggugcCguugc......................................   1       1   Mm1
....................................................guggguuguucggugcuUuugc......................................   1       1   Mm1
....................................................guggguuguucggugcuAuugc......................................   1       1   Mm1
....................................................guggguuguucggugcGguugc......................................   1       1   Mm1

ucaucucugcuuacaagag.............................................................................................   1       0   Mm2
...................cuauggcaccguagaauucacug......................................................................   5       0   Mm2
....................uauggcaccguagaauGc..........................................................................   1       1   Mm2
....................uauggcaccguagaauuc..........................................................................   11      0   Mm2
....................uauggcaccguagaauuca.........................................................................   4       0   Mm2
....................uauggcaccgAagaauucac........................................................................   1       1   Mm2
....................uauggcaccguagaauAcac........................................................................   1       1   Mm2
....................uauggcaccgCagaauucac........................................................................   1       1   Mm2
....................uauggcGccguagaauucac........................................................................   3       1   Mm2
....................uauggcaccguagaauucac........................................................................   202     0   Mm2
....................uauggcaccguagaauuGac........................................................................   1       1   Mm2
....................uauggcaccguagaauuAac........................................................................   3       1   Mm2
....................uaugCcaccguagaauucacu.......................................................................   1       1   Mm2
....................uauggcaccguaAaauucacu.......................................................................   1       1   Mm2
....................uauggcaccguaCaauucacu.......................................................................   1       1   Mm2
....................uauggcaccguUgaauucacu.......................................................................   2       1   Mm2
....................uauAgcaccguagaauucacu.......................................................................   5       1   Mm2
....................uauUgcaccguagaauucacu.......................................................................   1       1   Mm2
....................uauggcaccguagaauuAacu.......................................................................   53      1   Mm2
....................uauggUaccguagaauucacu.......................................................................   2       1   Mm2
....................uauggcaccguagaauAcacu.......................................................................   14      1   Mm2
....................uauggcUccguagaauucacu.......................................................................   1       1   Mm2
....................Cauggcaccguagaauucacu.......................................................................   1       1   Mm2
....................uauggcaccguagaauucCcu.......................................................................   1       1   Mm2
....................uauggcaccguagaauucacG.......................................................................   16      1   Mm2
....................uauggcaccgCagaauucacu.......................................................................   7       1   Mm2
....................uUuggcaccguagaauucacu.......................................................................   1       1   Mm2
....................uauggAaccguagaauucacu.......................................................................   1       1   Mm2
```

```
                    Mature                Star
ucaucucugcuuacaagagcuauggcaccguagaauucacuguguuuuuucagugguuguucggugcuguugcuuuugauguagauagcugcuguugucacuugugauuc
....................uauggcaccguagaauGcacu.....................................................................  13    1  Mm2
....................uauggcaccAuagaauucacu.....................................................................  1     1  Mm2
....................uGuggcaccguagaauucacu.....................................................................  2     1  Mm2
....................uauggcCccguagaauucacu.....................................................................  1     1  Mm2
....................uauggcaccguagaauucacu.....................................................................  5849  0  Mm2
....................uauggcaUcguagaauucacu.....................................................................  2     1  Mm2
....................uauggcaccUuagaauucacu.....................................................................  2     1  Mm2
....................uauggcaccguGgaauucacu.....................................................................  2     1  Mm2
....................uaugUcaccguagaauucacu.....................................................................  2     1  Mm2
....................uauggcaccgAagaauucacu.....................................................................  6     1  Mm2
....................uauggcGccguagaauucacu.....................................................................  5     1  Mm2
....................uauggcacUguagaauucacu.....................................................................  10    1  Mm2
....................uauggcaccguagaauucacA.....................................................................  3     1  Mm2
....................Nauggcaccguagaauucacu.....................................................................  2     1  Mm2
....................uaugAcaccguagaauucacu.....................................................................  1     1  Mm2
....................uauggcaccguagaauCcacu.....................................................................  16    1  Mm2
....................uauggcaccguagaauuGacu.....................................................................  37    1  Mm2
....................uauggcaccgGagaauucacu.....................................................................  8     1  Mm2
....................uauggNaccguagaauucacu.....................................................................  2     1  Mm2
....................uauCgcaccguagaauucacu.....................................................................  1     1  Mm2
....................uauggcaccguagCauucacu.....................................................................  1     1  Mm2
....................uauggcaccguagaauuUacu.....................................................................  24    1  Mm2
....................uauggcaccguagaauuUacug....................................................................  97    1  Mm2
....................uauggcaccguagaaCucacug....................................................................  1     1  Mm2
....................uauggcaccguGgaauucacug....................................................................  2     1  Mm2
....................uauggcaccgGagaauucacug....................................................................  21    1  Mm2
....................uaugUcaccguagaauucacug....................................................................  5     1  Mm2
....................uauggNaccguagaauucacug....................................................................  1     1  Mm2
....................uauggcaccgCagaauucacug....................................................................  20    1  Mm2
....................uauggcaccguagGauucacug....................................................................  1     1  Mm2
....................Nauggcaccguagaauucacug....................................................................  11    1  Mm2
....................uauggcacAguagaauucacug....................................................................  1     1  Mm2
....................Gauggcaccguagaauucacug....................................................................  1     1  Mm2
....................uauggcaccguagaauucacuU....................................................................  26    1  Mm2
....................uauggcaccguagUauucacug....................................................................  3     1  Mm2
....................uauggcaccguagaauucacuC....................................................................  8     1  Mm2
....................uauggcaccguagaauuNacug....................................................................  1     1  Mm2
....................uauggcaccAuagaauucacug....................................................................  8     1  Mm2
....................uauggcaGcguagaauucacug....................................................................  1     1  Mm2
....................uauggcNccguagaauucacug....................................................................  2     1  Mm2
....................uauggcaccguagaauGcacug....................................................................  43    1  Mm2
....................uauggcaccguUgaauucacug....................................................................  5     1  Mm2
....................uauggcaccguagaauucacuA....................................................................  16    1  Mm2
....................uauAgcaccguagaauucacug....................................................................  7     1  Mm2
....................uauggcaccguagaUuucacug....................................................................  4     1  Mm2
....................Aauggcaccguagaauucacug....................................................................  4     1  Mm2
....................uauggcaccguagaauAcacug....................................................................  52    1  Mm2
....................uaugCcaccguagaauucacug....................................................................  8     1  Mm2
....................uauggcaccguaUaauucacug....................................................................  10    1  Mm2
....................uauggcaccguagaauucGcug....................................................................  2     1  Mm2
....................uauggcaccguagaauucacCg....................................................................  1     1  Mm2
....................uauggcaccguagaauCcacug....................................................................  55    1  Mm2
....................uauUgcaccguagaauucacug....................................................................  3     1  Mm2
....................uauggcaccguagaaAucacug....................................................................  1     1  Mm2
....................uauggcaAcguagaauucacug....................................................................  2     1  Mm2
....................uauCgcaccguagaauucacug....................................................................  3     1  Mm2
....................uauggcaccguagaauucacGg....................................................................  37    1  Mm2
....................uauggcaccguagaauucacAg....................................................................  3     1  Mm2
....................uaCggcaccguagaauucacug....................................................................  3     1  Mm2
....................uauggcaccguCgaauucacug....................................................................  24    1  Mm2
....................uauggcacUguagaauucacug....................................................................  31    1  Mm2
....................uauggcaccguagaGuucacug....................................................................  2     1  Mm2
....................uNuggcaccguagaauucacug....................................................................  1     1  Mm2
....................uaugAcaccguagaauucacug....................................................................  3     1  Mm2
....................uUuggcaccguagaauucacug....................................................................  7     1  Mm2
....................uauggcGccguagaauucacug....................................................................  15    1  Mm2
....................uauggcaccUuagaauucacug....................................................................  7     1  Mm2
....................uGuggcaccguagaauucacug....................................................................  7     1  Mm2
....................uauggcaccguagaauuAacug....................................................................  174   1  Mm2
....................uauggcaccguagaauucaUug....................................................................  3     1  Mm2
```

Mature **Star**

```
ucaucucugcuuacaagagcuauggcaccguagaauucacuguguuuuuuucaguggguuguucggugcuguugcuuuugauguagauagcugcuguugucacuugugauuc
....................uauggUaccguagaauucacug......................................................................   12      1   Mm2
....................uauggcaccguagaauucacug......................................................................   20205   0   Mm2
....................uauggcaccguagaauuGacug......................................................................   81      1   Mm2
....................uauggcaccgAagaauucacug......................................................................   27      1   Mm2
....................uaGggcaccguagaauucacug......................................................................   2       1   Mm2
....................uauggcaccguagaauucCcug......................................................................   2       1   Mm2
....................uauggcaUcguagaauucacug......................................................................   2       1   Mm2
....................uauggcaccguagaauucUcug......................................................................   2       1   Mm2
....................uauggcaccguaAaauucacug......................................................................   1       1   Mm2
....................uauggcCccguagaauucacugu.....................................................................   1       1   Mm2
....................uauggcaccguagaauucacuUu.....................................................................   1       1   Mm2
....................uauggcaccguagaauuAacugu.....................................................................   7       1   Mm2
....................uauggcaccguagaaCucacugu.....................................................................   1       1   Mm2
....................uauggcaccguUgaauucacugu.....................................................................   1       1   Mm2
....................uauggAaccguagaauucacugu.....................................................................   1       1   Mm2
....................uauggcaccguagaauuGacugu.....................................................................   4       1   Mm2
....................uauggcaccguagaauucacugu.....................................................................   901     0   Mm2
....................uauggcaccguagaauucacugA.....................................................................   205     1   Mm2
....................uauggcaccguagaauAcacugu.....................................................................   2       1   Mm2
....................uauggcGccguagaauucacugu.....................................................................   2       1   Mm2
....................uauggcaccguagaauucacugG.....................................................................   20      1   Mm2
....................uauggcaccguagaauucacugC.....................................................................   1       1   Mm2
....................uauggcacUguagaauucacugu.....................................................................   2       1   Mm2
....................uauggcaccguagaauGcacugu.....................................................................   1       1   Mm2
....................uauggcaccguagaauucacAgu.....................................................................   1       1   Mm2
....................uaNggcaccguagaauucacugu.....................................................................   1       1   Mm2
....................uauggcaccguagaauuUacugu.....................................................................   6       1   Mm2
....................uauggcaccgAagaauucacugu.....................................................................   1       1   Mm2
....................uaGggcaccguagaauucacugu.....................................................................   1       1   Mm2
....................uauAgcaccguagaauucacugu.....................................................................   1       1   Mm2
....................uauggcaccguagaauucacuguA....................................................................   4       1   Mm2
....................uauggcaccguagaauucacuguU....................................................................   19      1   Mm2
....................uauggcaccguagaauucacugugG...................................................................   1       1   Mm2
....................uauggcaccguagaauucacuguUu...................................................................   1       1   Mm2
....................uauggcaccguagaauucacuguUuuuuuuu.............................................................   1       1   Mm2
.....................auggcaccguagaauucac........................................................................   1       0   Mm2
.....................auggcaccguagaauucacu.......................................................................   5       0   Mm2
.....................auggcaccguaAaauucacu.......................................................................   1       1   Mm2
.....................auggcaccguagaauucGcug......................................................................   1       1   Mm2
.....................auggcaccguagaauucacug......................................................................   35      0   Mm2
.....................auggcaccguagaauucacugu.....................................................................   1       0   Mm2
.....................auggcaccguagaauucacugA.....................................................................   1       1   Mm2
......................uggcaccguagaauucacug......................................................................   1       0   Mm2
..........................................uguuuuuuucUguggguuguucggugcuguugc.....................................   1       1   Mm2
.....................................................guggguuguucggugcuguug......................................   2       0   Mm2
.....................................................guggguuguucggugcuAuugc.....................................   2       1   Mm2
.....................................................guggguuguucggugcuguugc.....................................   68      0   Mm2
.....................................................guggguuguucggugcuguugcu....................................   5       0   Mm2
.....................................................guggguuguucggugcuguugcA....................................   2       1   Mm2
.....................................................guggguuguucggugcuguugcG....................................   1       1   Mm2
.....................................................guggguuguucggugcuguugcuu...................................   1       0   Mm2

....................uauggcaccguagaauuc..........................................................................   3       0   Mm3
....................uauggcaccguagaauuca.........................................................................   3       0   Mm3
....................uauggcaccguagaauucac........................................................................   36      0   Mm3
....................uauggcaccguagaauucacu.......................................................................   783     0   Mm3
....................uauggcaccguagaauCcacu.......................................................................   1       1   Mm3
....................uauggcaccguagaauuAacu.......................................................................   3       1   Mm3
....................uauggcaccguagaauAcacu.......................................................................   2       1   Mm3
....................uauggcaccguagGauucacu.......................................................................   1       1   Mm3
....................uauggcaccUuagaauucacu.......................................................................   1       1   Mm3
....................uauggcaccguagaauucacA.......................................................................   2       1   Mm3
....................uauggcaccguagaauucacG.......................................................................   3       1   Mm3
....................uauggcaccguGgaauucacu.......................................................................   1       1   Mm3
....................uauggcaccguagaauuUacu.......................................................................   5       1   Mm3
....................uauggcaccgCagaauucacu.......................................................................   1       1   Mm3
....................uauggcGccguagaauucacu.......................................................................   2       1   Mm3
....................uauggUaccguagaauucacu.......................................................................   1       1   Mm3
....................uauggcaccguagaauCcacug......................................................................   10      1   Mm3
....................uauggcaccguagaauuGacug......................................................................   10      1   Mm3
```

```
                    Mature                           Star
ucaucucugcuuacaagagcuauggcaccguagaauucacuguguuuuuuucaguggguuguucggugcuguugcuuuugauguagauagcugcuguugucacuugugauuc
....................uauggcacUguagaauucacug......................................................................   4      1   Mm3
....................uGuggcaccguagaauucacug......................................................................   2      1   Mm3
....................uauggcaccguagaauucacug......................................................................   2731   0   Mm3
....................uauggcaccguagaGuucacug......................................................................   1      1   Mm3
....................uauggcaccguagaauAcacug......................................................................   8      1   Mm3
....................Nauggcaccguagaauucacug......................................................................   1      1   Mm3
....................uauggcaccguagaauGcacug......................................................................   6      1   Mm3
....................uauggcaccgGagaauucacug......................................................................   6      1   Mm3
....................uauggcGccguagaauucacug......................................................................   2      1   Mm3
....................uaugUcaccguagaauucacug......................................................................   1      1   Mm3
....................uauggcaccAuagaauucacug......................................................................   3      1   Mm3
....................uauggcaccgCagaauucacug......................................................................   4      1   Mm3
....................uauggcaccguaUaauucacug......................................................................   1      1   Mm3
....................uauggcaccUuagaauucacug......................................................................   1      1   Mm3
....................uauggcaccguGgaauucacug......................................................................   2      1   Mm3
....................uauggUaccguagaauucacug......................................................................   1      1   Mm3
....................uauggcaccguagaauuAacug......................................................................   28     1   Mm3
....................uauUgcaccguagaauucacug......................................................................   1      1   Mm3
....................uauggcaccguagaCuucacug......................................................................   1      1   Mm3
....................uauggcaccguagaauucacuA......................................................................   1      1   Mm3
....................uauggcaccguagaauuUacug......................................................................   15     1   Mm3
....................uauggcaccguagaauucacuU......................................................................   4      1   Mm3
....................uauggNaccguagaauucacug......................................................................   1      1   Mm3
....................uauggcaccguagaauucacGg......................................................................   4      1   Mm3
....................uauggcaccgAagaauucacug......................................................................   9      1   Mm3
....................uUuggcaccguagaauucacug......................................................................   1      1   Mm3
....................uauggcNccguagaauucacug......................................................................   1      1   Mm3
....................uauggcaccguagaauucacCg......................................................................   1      1   Mm3
....................uauggcaccguUgaauucacug......................................................................   1      1   Mm3
....................uauggcUccguagaauucacug......................................................................   1      1   Mm3
....................uauAgcaccguagaauucacug......................................................................   3      1   Mm3
....................uauggcaccguCgaauucacug......................................................................   4      1   Mm3
....................uauggcaccguagaUuucacug......................................................................   1      1   Mm3
....................uauggcaUcguagaauucacug......................................................................   1      1   Mm3
....................uauggcaccguagaauucacugC.....................................................................   1      1   Mm3
....................uauggcaccguagaauucacugA.....................................................................   41     1   Mm3
....................uauggcaccguagaauucacugu.....................................................................   138    0   Mm3
....................uauggcacUguagaauucacugu.....................................................................   1      1   Mm3
....................uauggcaccguagaauAcacugu.....................................................................   1      1   Mm3
....................uauggcaccguagaauuUacugu.....................................................................   1      1   Mm3
....................uauggcaccguagaauucacugG.....................................................................   4      1   Mm3
....................uauggcaccguagaauGcacugu.....................................................................   1      1   Mm3
....................uauggcaccguagaauuAacugu.....................................................................   1      1   Mm3
....................uauggcaccguagaauCcacugu.....................................................................   1      1   Mm3
....................uauggcaccgAagaauucacugu.....................................................................   1      1   Mm3
....................uauggcaccguagaauucacuguU....................................................................   21     1   Mm3
....................uauggcaccguagaauucacuguA....................................................................   2      1   Mm3
.....................auggcaccguagaauucacu.......................................................................   2      0   Mm3
.....................auggcaccguagaauucacug......................................................................   5      0   Mm3
.....................................................guggguuguucggugcuguugc.....................................   18     0   Mm3
.....................................................guggguCguucggugcuguugc.....................................   1      1   Mm3
.....................................................guggguuguucgguAcuguugc.....................................   1      1   Mm3
.....................................................guggguuguucggugcuguugcu...................................   3      0   Mm3
.....................................................guggguuguucggugcuguugcuu..................................   1      0   Mm3
.....................................................guggguuguucggugcuguugcuuu.................................   1      0   Mm3
...........................................................................uuuugauguagauagcugcu.................   1      0   Mm3
```
